# Variation in red blood cell transfusion thresholds in critically ill patients

**DOI:** 10.1186/cc13296

**Published:** 2014-03-17

**Authors:** K Wilton, R Fowler, T Walsh, J Lacroix, J Callum

**Affiliations:** 1 Sunnybrook Hospital, Toronto, Canada

## Introduction

Restrictive transfusion practice in recent randomized trials and systematic reviews continues to have favorable outcomes [1]. Despite this, substantial variability in transfusion practice persists [2]. It is important to identify contemporary variability, and which patient, provider, and institutional factors drive this variability. Therefore, we performed a retrospective analysis hypothesizing that red blood cell (RBC) transfusion practice remains variable and is influenced by patient characteristics and institutional transfusion culture.

## Methods

We performed a multicenter retrospective analysis within the ongoing prospective randomized, Age of Blood Evaluation (ABLE) trial. The patient population included patients admitted to the ICU with an anticipated length of invasive or non-invasive mechanical ventilation >48 hours and who required a first RBC transfusion during the first 7 days of ICU admission. As of March 2013, completed and verified data from 45 sites in Canada, France, and the UK were included. Sites with at least 12 patients were included in site analysis. The primary outcome is the association of enrolling centers on the median hemoglobin prior to transfusion.

## Results

As of March 2013, there were 1,288 patients randomized in the ABLE study. The median patient age was 63 years (IQR 50 to 74). The majority of patients were emergent admissions (97%) with nonoperative diagnoses (85%), including respiratory illness (27%) and sepsis (18%). Bleeding was infrequent (0.4%). At least one comorbidity was common (43%), frequently significant cardiac disease (14%) and diabetes (12%). There is significant variability in the median pretransfusion hemoglobin across different sites (*P *< 0.05). The median pre-transfusion hemoglobin by site was 75 g/l (IQR 71 to 77). Six of 24 sites had a significantly higher, and four had a significantly lower, median transfusion threshold compared with the median. Significant variability in the median pre-transfusion hemoglobin remained in multivariate analysis, after adjustment for age and type of ICU. Conclusion There was a 16 g/l difference in median hemoglobin between the lowest and highest site and wide inter-site variation. These differences may be due to transfusion education, monitoring and blood-bank enforcement practices. Site, not country, remains a significant predictor of transfusion. Future directions in transfusion best practices should focus on local transfusion culture. Significant and nonevidence-based variability persists in transfusion thresholds for critically ill patients.
